# Effects of Aerobic Exercise as Add-On Treatment for Inpatients With Moderate to Severe Depression on Depression Severity, Sleep, Cognition, Psychological Well-Being, and Biomarkers: Study Protocol, Description of Study Population, and Manipulation Check

**DOI:** 10.3389/fpsyt.2019.00262

**Published:** 2019-04-25

**Authors:** Christian Imboden, Markus Gerber, Johannes Beck, Anne Eckert, Uwe Pühse, Edith Holsboer-Trachsler, Martin Hatzinger

**Affiliations:** ^1^Psychiatric Services Solothurn, Solothurn, Switzerland; ^2^Medical Faculty, University of Basel, Basel, Switzerland; ^3^Private Clinic Wyss, Münchenbuchsee, Switzerland; ^4^Department of Sport, Exercise and Health, University of Basel, Basel, Switzerland; ^5^Psychiatric University Hospital, University of Basel, Basel, Switzerland; ^6^Private Clinic Sonnenhalde, Riehen, Switzerland

**Keywords:** aerobic exercise, depression, treatment, cognition, sleep, brain-derived neurotropic factor, cortisol

## Abstract

**Background:** Aerobic exercise (AE) may be a non-pharmacological strategy to improve depression treatment and lessen the burden of somatic comorbidity of depression. Only few studies have examined the effect of AE as an add-on treatment for moderate to severe depression in an inpatient setting, and most studies have focused on depression severity and cardiovascular parameters. The purpose of the present article is to present the study protocol, to provide information about the assessed study population, and to perform a manipulation check in order to examine whether the intervention program was properly implemented.

**Methods:** We conducted a randomized controlled trial in two centers comparing 6 weeks of AE to a placebo control intervention (stretching) as an add-on to standardized inpatient treatment of moderate to severe depression. Besides depression severity, several other psychological and biological variables were measured such as salivary cortisol, brain-derived neurotropic factor, cognitive tests, and polysomnography. To evaluate long-term effects of the intervention, we also scheduled a follow-up 6 months after completion of the study intervention.

**Results:** Forty-five patients were randomized to either AE (*n* = 23) or the placebo intervention (*n* = 22); 36 patients completed the 6-week intervention. In the AE group, 65% completed all 18 training sessions. Patients who were less physically active prior to admission were less likely to complete the study. With regard to energy expenditure, mean kcal/kg/week was 16.4 kcal/kg/week (range: 13.8–17.7), coming close to the targeted dose of 17.5 kcal/kg/week.

**Conclusions:** Overall, patients showed good adherence to the intervention protocol despite at least moderate depression severity. However, the dropout rate suggests that depressed inpatients may need special support to adhere to a structured exercise intervention program. This study will add evidence on the effects of AE as an add-on to inpatient treatment of moderate to severe depression. Besides antidepressant effects, potentially beneficial effects of AE on a broad array of further variables associated with depression will be evaluated.

**Clinical Trial Registration:** www.ClinicalTrials.gov, identifier NCT02679053.

## Introduction

According to the World Health Organization (WHO), unipolar depression is considered the most disabling condition concerning disability-adjusted life years in middle- to high-income countries (1). Of all brain disorders, depression causes the highest economic burden in Switzerland (2). With approximately 30% of depressed patients meeting criteria for treatment-resistant depression (3) and a lack of new pharmacological targets besides the well-known monoaminergic compounds, there is a need for new treatment strategies for depression.

Aerobic exercise (AE) may be one such a strategy as it has proven to be an effective treatment for mild to moderate depression in several meta-analyses with small to moderate effect sizes (4, 5). However, if AE is compared to a comparator–intervention and if only methodologically sound studies are included in the pooled analysis, the effect size associated with AE is considerably lower. Furthermore, there is still a lack of methodically sound studies incorporating a large array of neurobiological factors such as sleep, hypothalamic–pituitary–adrenal (HPA) axis, and brain-derived neurotropic factor (BDNF). While there is a rather large body of evidence on the effects of AE in mild to moderate depression in outpatients, studies evaluating the effects of AE in more severely depressed patients, especially inpatients, are still scarce, and more research is needed (6). Schuch et al. (7) showed a significant group × time interaction effect for symptom severity in depressed inpatients randomized to either AE or stretching activities. However, in their study, the mean duration of the hospital stay was only 3 weeks, and the sample consisted of 75% female participants. Another study conducted by Kerling et al. (8) compared a 6-week exercise program to treatment as usual (TAU) in depressed inpatients and found that patients participating in the AE group were more likely to show treatment response and showed significantly better cardiorespiratory fitness, waist circumference, and High Density Lipoprotein (HDL) cholesterol than patients in the TAU group. In a study by Legrand and Neff (9), adding AE during the first 2 weeks of inpatient treatment for depression to pharmacological treatment showed a significant reduction of depressive symptoms assessed with the Beck Depression Inventory (BDI) for groups performing either AE or stretching activities, but not for the TAU group. Moreover, a stronger effect was found for the AE group compared to the stretching group. The study, however, included no information on medication, and there was no follow-up assessment to examine whether the effects were maintained beyond patients’ stay at the clinic. Shachar-Malach et al. (10) carried out a preliminary trial in Israel with a relatively small sample (*n* = 12). In their study, 3 weeks of AE was associated with a significant reduction in symptom severity, which was not the case for the control condition (stretching activities). In contrast, Buschert et al. (11) compared 3 to 4 weeks of AE to occupational therapy in a sample of 38 depressed inpatients and found no beneficial effect of AE on depressive symptoms. In summary, there is a current trend toward comparing AE to an active control group that usually consists of stretching activities rather than TAU or a waiting-list control group. The few existing studies evaluating AE as an add-on-treatment for depressed inpatients vary in duration. There is little information regarding treat­ments other than pharmacotherapy such as diagnosis-specific psychotherapy or creative and body-oriented therapy, thus reflecting the internationally varying standards of inpatient care and its role in the health care system. Therefore, genera­lizability of findings from one inpatient setting to others is difficult.

Additionally, few studies on AE and depression examined factors other than symptom severity or cardiovascular parameters. Schuch et al. (12) evaluated the effect of AE in inpatients on serum BDNF (sBDNF) and found no additional effect; however, they observed a decrease in markers associated with oxidative stress. Moreover, dysfunctional HPA axis is an important biomarker of depression (13), and its normalization could predict antidepressant response as well as long-term course of the disease (14). Gerber et al. (15) performed a study with healthy college students showing that subjects who exercised more often showed a more favorable pattern of cortisol secretion during a social stress test. While there is evidence on beneficial effects of regular AE on sleep quality in healthy subjects (16, 17) and chronic insomniacs (18), exercise studies that incorporate objective sleep measures such as polysomnography and subjective sleep quality in depressed patients are still scarce. Since sleep is an important symptom of depression, predicting relapse if persistent at remission (19), potentially beneficial effects of AE on sleep could play an important role in depression treatment.

Finally, there is a growing body of evidence supporting a beneficial effect of AE on cognitive symptoms associated with depression such as impairments of working memory and executive function (11, 20–23). Since cognitive symptoms of depression often are a persisting problem not being properly targeted by antidepressants (24, 25), this may be another promising benefit of AE. We therefore argue that the examination of cognitive performance indicators needs to be incorporated more systematically into studies on AE in depression.

## Study Objectives

Given the background presented above, the primary objective of our study was to evaluate the antidepressant effect of a 6-week AE program as an add-on treatment in moderately to severely depressed inpatients treated with a multimodal regimen. Secondary objectives of the study were to measure the effects of AE on cognition, subjective and objective sleep parameters, HPA axis including adrenocortical reactivity to psychosocial stress, BDNF, tumor necrosis factor-alpha (TNF-alpha), and various psychological variables. By studying a broad array of biological variables, we aim to advance knowledge about differential effects of AE as an add-on treatment and the neurobiological background of inpatients with moderate to severe depression.

The purpose of the present article is to present the study protocol, to provide information about the assessed study population, and to perform a manipulation check in order to examine whether the intervention program was properly implemented.

## Materials and Methods

### Study Design and Population

We conducted a two-center, two-armed randomized controlled trial (RCT) with participants allocated to AE or a standardized stretching and mobility program (control group). Blinding of patients was achieved by informing them that two different exercise programs were to be compared, but not telling them which intervention was considered the intervention or control condition. All assessors of psychological variables as well as local study coordinators were fully blinded to group allocation. Patients who were hospitalized in the depression wards of the Psychiatric Services Solothurn or the Psychiatric University Clinics Basel and suffered from moderate to severe depression [Hamilton Depression Rating Scale 17 (HDRS17) > 16] were considered for study inclusion. Assuming a moderate effect (*f* = 0.25) of the intervention on the primary outcome (depression severity), we performed a power analysis for repeated-measures analyses of variance (ANOVAs) to estimate the minimal sample size [using G*Power 3.1; alpha error probability was set at .05, power at .90, and correlations among measures at .50, using the option “within-between”(see **Figure S1**)]. Based on this analysis, the minimally required sample size was *N* = 36 participants. We therefore aimed at including 40 patients in the study with a similar group and sex distribution. Because we expected a maximal dropout of 20%, we decided to approach and recruit a maximum of 50 participants for the study. Recruitment took place consequently between October 2013 and January 2016. Randomization was achieved by drawing lots for AE or control condition at a 1:1 ratio. To achieve a balanced sex distribution, lots were drawn separately for males and females. Randomization was carried out by the administrative centers of the clinics, which then informed the external exercise coaches who supervised all training sessions. Medical staff in the wards had no knowledge of the group allocation. All data assessments were performed by trained research officers, who were blinded to group allocation.

### Ethics Approval and Consent to Participate

This study was carried out in accordance with the recommendations of APA ethical standards with written informed consent from all subjects. All subjects gave written informed consent in accordance with the Declaration of Helsinki. Ethical approval was obtained by the following ethics committees: i) Ethics Committee of Both Basel (EKBB) in Basel, Switzerland (reference no. 62/13, obtained on May 6th, 2013), and ii) Ethics Committee Aargau/Solothurn in Aarau, Switzerland (reference no. 2013/029, obtained May 21st, 2013). Today, both ethics committees are represented by the newly formed Ethics Committee of Northwestern and Central Switzerland (EKNZ) in Basel, Switzerland. To assess an inflammatory parameter, we added measurement of TNF-alpha after completing the study; therefore, we obtained a protocol amendment by the EKNZ (reference no. PB_2016-02488, obtained November 14th, 2016). The study was retrospectively registered on ClinicalTrials.gov in February 2016 (identifier: NCT02679053).

### Inclusion and Exclusion Criteria

Participants had to meet the following inclusion criteria: a) aged >18 or <61 years, b) an inpatient in respective depression wards, c) ICD-10 (International Classification of Diseases, Tenth Revision) diagnosis of depression (first episode, recurrent or bipolar; F32, F33, F31), d) a score of >16 on the HDRS17, and e) written informed consent.

Participants were not eligible for the study if they met one of the following criteria: a) presence of a somatic condition not permitting regular AE, b) body mass index (BMI) > 35 kg/m^2^, c) pregnancy at baseline, d) acute suicidal ideation,e) comorbid substance dependency (except nicotine), f) comorbid major psychiatric disorder, and g) regular participation in high-intensity exercise activities.

#### Intervention and Control Conditions

The intervention group was scheduled for supervised AE on indoor bicycles three times per week for six consecutive weeks at a target heart rate (HR) of 60–75% of maximal HR (HRmax) monitored with Polar™ RS800CX. HRmax was computed using the age formula [220 – age (in years)]. The weekly amount of energy expended through AE was defined by calories on the basis of 17.5 kcal per kg bodyweight, as suggested by Dunn et al. (26).

For the control condition, we developed a program consisting of basic coordination and stretching techniques for all major muscle groups using a medium-strength Theraband®, a gymnastics ball (diameter, 65 cm), and juggling balls. Supervisors ensured that all activities were kept at low intensity. The activities were changed regularly during the intervention period to keep the intervention interesting for participants. The control intervention also took place three times per week for six consecutive weeks.

All intervention sessions took place in the late afternoon (between 4 and 6 p.m.) for approximately 40 to 50 min. All patients underwent standard inpatient treatment consisting of pharmacological treatment according to Swiss national standards (27) and individual and group psychotherapy supported by an array of creative group therapies. However, participants were asked not to engage in any additional vigorous exercise activities during their stay at the hospital. Pharmacological treatment was limited to antidepressant treatment with selective serotonin reuptake inhibitors (SSRIs) or selective serotonin–norepinephrine reuptake inhibitors (SNRI) and lithium as an augmentation strategy. Antidepressant combination therapy, tricyclic antidepressants, MAO inhibitors, and antipsychotics were not allowed during the study period. An exception was quetiapine for sedation and augmentation treatment.

### Measures

An overview of all measures is provided in **Table 1**. In the following sections, more specific information is provided about all assessed variables, at baseline, after 1 week, after 2 weeks, after completion of the intervention, and at follow-up. All data assessments took place at the clinics where the patients received treatment.

**Table 1:** Overview of study design and measurements.

		STUDY PERIOD					
TIME POINT		Enrollment	Allocation	Post-allocation			
			Baseline	+1 week	+2 weeks	Post (+6 weeks)	Follow-up (+6 months)
ENROLLMENT							
Eligibility screening		X					
Demographic data		X					
Informed consent		X					
Group allocation			X				
INTERVENTIONS							
Aerobic exercise condition				6 weeks of aerobic exercise (17.5 kcal/kg/week)			
Control condition				6 weeks of standardized control intervention (including low-intensity stretching and mobility activities)			
MEASURES							
Physical and psychiatric history		X					
Psychiatric diagnosis (ICD-10)		X					
Physical examination		X					
Queens Step Test			X	Weekly			
ECG		X					
BP, HR		X				X	X
BMI		X				X	X
PSG			X			X	
Cognition (TAP V. 2.3):			X			X	X
-Working memory							
-Alertness							
-Flexibility							
-Go/no-go							
-Divided attention							
Cortisol awakening response (CAR)			X		X	X	X
Trier Social Stress Test (TSST)			X			X	
sBDNF			X		X	X	X
TNF-alpha			X		X	X	
**Rating scales:**	HDRS17	X		X	X	X	X
****	BDI	X		X	X	X	X
****	SCL-90R		X			X	X
****	PSQI		X			X	X
****	MTQ18		X			X	X
****	PSDQ		X			X	
****	FEPS II		X			X	X
****	IPAQ-SF		X				X

ICD-10, International Classification of Diseases, Tenth Revision. ECG, electrocardiogram. BP, blood pressure. HR, heart rate. BMI, body mass index. PSG, polysomnography. TAP, Testbatterie zur Aufmerksamkeitsprüfung; English: battery of tests for the assessment of attention. sBDNF, serum brain-derived neurotropic factor. TNF-alpha, tumor necrosis factor-alpha. HDSR17, Hamilton Depression Rating Scale 17. BDI, Beck Depression Inventory. SCL-90R, Symptom Checklist-90. PSQI, Pittsburgh Sleep Quality Index. MTQ18, Mental Toughness Questionnaire (18-item version). PSDQ, Physical Self-Description Questionnaire. FEPS II, Fragebogen zur Erfassung allgemeiner und spezifischer Persönlichkeitsmerkmale Schlafgestörter; English: questionnaire for general and specific personality traits of insomniacs. IPAQ-SF, International Physical Activity Questionnaire (Short Form).

#### Baseline Data Assessment

*Basic and demographic data:* We assessed age, smoking status (cigarettes per day and pack years), duration of current depressive episode, number of prior depressive and (hypo)maniac episodes, age of onset of depression, and educational status. Additionally, all patients underwent a physical examination including electrocardiogram (ECG), blood pressure (BP), resting HR, as well as body weight and height. To measure the level of physical activity prior to hospital admission, we used the short form of the International Physical Activity Questionnaire (IPAQ-SF) (28). Symptom severity of depression was measured with the 17-item Hamilton Depression Rating Scale (29) and the 21-item BDI (30).

*Sleep:* Subjective sleep quality was measured with the Pittsburgh Sleep Quality Index (PSQI) (31). To assess dysfunctional cognitive processes involved in the exacerbation and perpetuation of insomnia, the Fragebogen zur Erfassung allgemeiner und spezifischer Persönlichkeitsmerkmale Schlafgestörter (FEPS II; English: questionnaire for general and specific personality traits of insomniacs) (32) was administered. The FEPS II is a German questionnaire composed of two subscales labeled “focussing” and “rumination.” “Focussing” refers to a person’s tendency to continuously think about sleep-associated difficulties, whereas “rumination” describes a person’s proneness to worry about unresolved problems prior to falling asleep. To measure objective sleep, we performed a polysomnography (PSG) during one night with Compumedics somtéPSG [electroencephalography (EEG) canals C3 and C4, electrooculography (EOG), electromyography (EMG), and ECG] for sleep staging.

*HPA axis:* To measure HPA-axis activity, we assessed cortisol awakening response (CAR) by measuring salivary cortisol immediately after waking up and 10, 20, and 30 min later (33). To measure reactivity to experimentally induced psychosocial stress, patients were asked to participate in the Trier Social Stress Test (TSST) (34), consisting of a standardized (free speech) task (job interview, application for a promotion or a price) and a mental arithmetic task (counting backward in steps of 13 from a 4-digit number), each lasting for 5 min. Patients had 5 min to prepare themselves for the tasks. The test took place in front of a jury of two persons who were unknown to the patients; patients were told that the test would be videotaped. Salivary cortisol was obtained at −20, −5, +1, +5, +10, +20, +30, +45, and +60 min prior to/after the test. TSST took place at exactly 2 p.m. to control for circadian variations of cortisol levels. Since the TSST was scheduled a second time after 6 weeks, patients were only debriefed about the true nature of the test and the fact that no videos were recorded after completion of the second TSST session.

*Cognition:* Cognitive variables were measured with the TAP V 2.3 (Testbatterie zur Aufmerksamkeitsprüfung; English: battery of tests for the assessment of attention), a computerized testing suite to conduct several cognitive tests focusing on attention, prompting patients to press a button at various conditions as fast as possible. We conducted the following subtests: alertness, working memory, divided attention, flexibility, and go/no-go.

*Laboratory parameters:* sBDNF and TNF-alpha were measured in serum samples [ethylenediaminetetraacetic acid (EDTA) blood]. Due to diurnal variations of sBDNF (35), all samples were obtained at the same time in the morning (8 a.m.).

*Assessment of further psychological constructs:* Mental  toughness, a psychological construct related to positive stress management that is known to be associated with good psychological well-being and resilience against stress (36), was measured by the 18-item short form of the Mental Toughness Questionnaire (MTQ18) (37). Athletic abilities, self-esteem, and health were measured with a German version (38) of the Physical Self-Description Questionnaire (PSDQ) (39). General psychological and somatic symptoms were assessed with the Symptom Checklist-90 (SCL-90R) (40).

#### +1 Week

After the first week of intervention, information regarding depression severity was assessed with the BDI and HDRS17, in order to identify early responders.

#### +2 Weeks

After 2 weeks of exercise, we measured CAR, TNF-alpha, and sBDNF, in addition to depression severity (assessed with the BDI and HDRS17).

#### Post Intervention (+6 Weeks)

After completion of the intervention, we measured BDI, HDRS17, body weight, BP, HR, sBDNF, TNF-alpha, CAR, PSQI, FEPSII, MTQ18, PSDQ, and SCL-90R. A second PSG was performed for sleep staging. The same cognitive performance tests (TAP) were carried out as at baseline, and a second TSST was performed with slightly modified tasks (41). All medication taken on a fixed schedule was registered.

#### Follow-Up (+6 Months)

After 6 months, we employed the following instruments for the follow-up data assessment: BDI, HDRS17, IPAQ, body weight, BP, HR, sBDNF, CAR, PSQI, FEPSII, MTQ18, and SCL-90R. TAP tests were repeated.

#### Measurements During Exercise Sessions

During each training session, we assessed duration of exercise, mean heart rate (HRmean), expended kilocalories (kcal) as reported by Polar HR monitor, and perceived exertion by the Borg scale (1–20, 42). At baseline and at the end of every week, participants from the intervention group performed a Queens Step Test (43, 44) to monitor improvements in their cardiorespiratory fitness.

### Data Collection and Management

After completion of the data assessment, all personal data of the patients were encoded (each patient received a project ID number), so that it was no longer possible to identify the patients. The coding list is stored at a safe place. Data were entered into an SPSS file. Statistical analyses were performed using SPSS. Data were only used for scientific purposes and were discarded after completion of the laboratory analyses. Paper records of the study are only accessible to the main investigators and are kept in locked cupboards. After 5 years, all records will be destroyed. Only authorized researchers have access to the data entered into the computerized files.

### Data Analysis

To test whether variables were normally distributed, results of the Kolmogorov–Smirnov and Shapiro–Wilk tests were inspected. To examine whether baseline differences existed between the intervention and control groups, in case of normally distributed variables, ANOVAs were conducted, and descriptive statistics are reported as M and SD. In the case of non-normally distributed variables, nonparametric tests were used such as independent-samples median test, independent-samples Mann–Whitney U test, and independent-samples Kruskal–Wallis test. For non-normally distributed variables, the median and range (min; max) are reported. For categorical variables, χ^2^ tests were used to examine group differences, and *n* and % are reported as descriptive statistics.

Furthermore, descriptive statistics (*M*, *SD*, median, range, skewness, kurtosis) are presented to examine whether participants in the intervention group adhered to the targeted AE protocol.

When presenting the main findings of the study in future publications, changes in outcome variables over the three time points will be analyzed using repeated-measures analysis of covariance (ANCOVA), with a between-subject factor group (intervention vs. control group) and a within-subject factor time (baseline, post, follow-up). In case of non-normally distributed outcomes, variables will be logarithmized to achieve a normal distribution. If significant group or time interactions are present, Bonferroni-adjusted *post hoc* tests will be performed to identify individual differences. Statistical significance level will be defined at an alpha level of .05. Medication and smoking will be taken into consideration when assessing the impact of the intervention on blood parameters, as these factors may impair some of these outcomes.

## Results

### Sample Description

**Figure 1:**
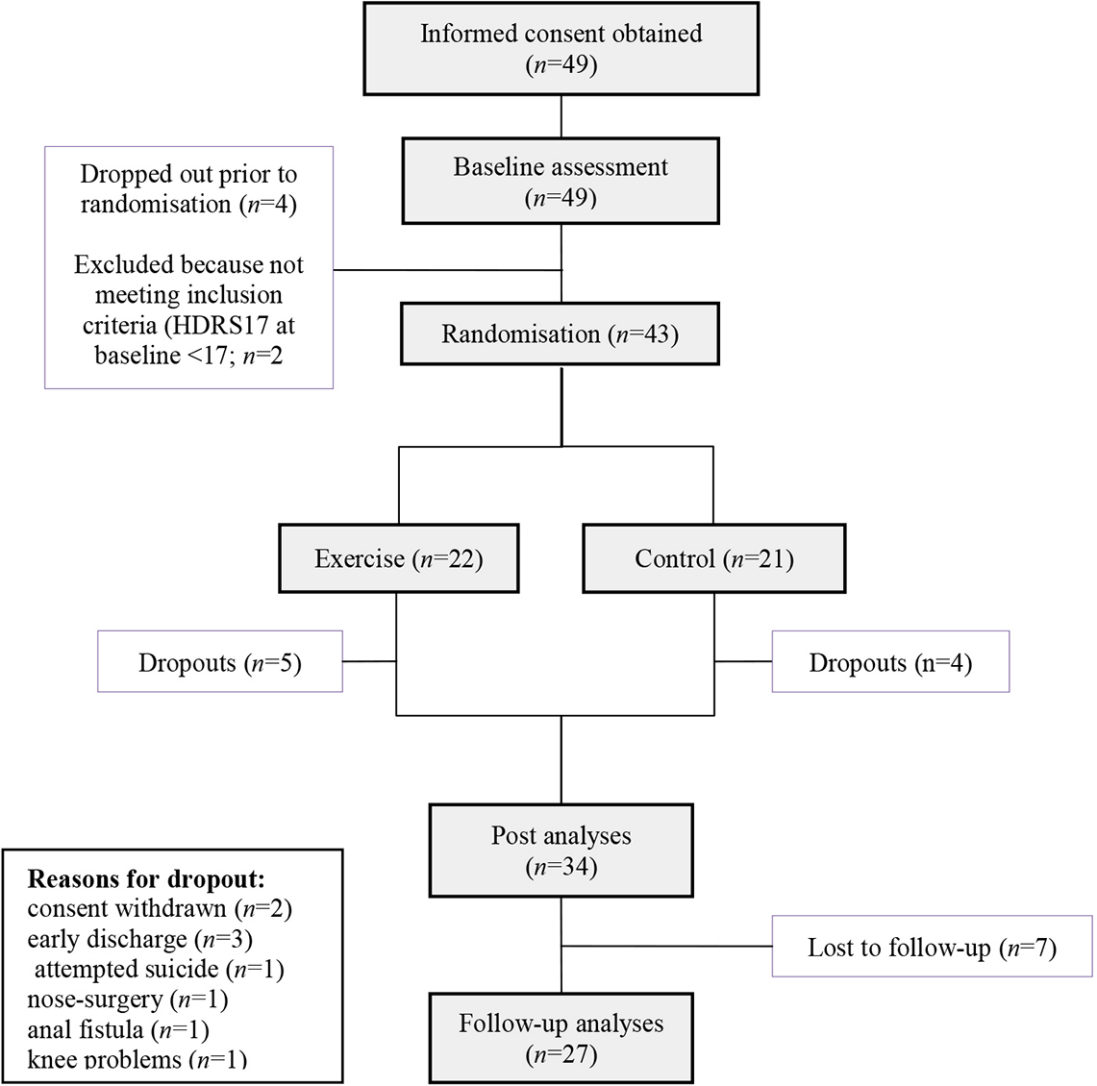
Participant flowchart.

Between October 2013 and January 2016, informed consent was obtained from 49 patients. These patients took part in the baseline data assessment. Four patients withdrew consent prior to randomization. Two patients had to be excluded because they did not meet inclusion criteria at baseline concerning symptom severity (HDRS17 < 17). The last follow-up visit took place in August 2016. Forty-three patients were randomized to either AE (*n* = 22) or the control intervention (*n* = 21). During the course of the intervention, a total of 9 patients (20%; AE group: *n* = 5, control group: *n* = 4) withdrew consent (*n* = 2) or had to be excluded due to other reasons (*n* = 3 patients were discharged early, *n* = 1 patient attempted suicide, *n* = 1 patient had to undergo nasal surgery, *n* = 1 patient developed knee problems, and *n* = 1 patient developed an anal fistula). The attempted suicide was rated as a severe adverse event and reported to the local ethics committee with no consecutive consequences for the study other than a more rigorous assessment of suicidal behavior prior to study inclusion. The anal fistula occurred in the control group, and there was no reason to believe that it was caused by the study intervention. In total, *n* = 34 of the patients who met the study inclusion criteria completed the intervention. During the follow-up period, *n* = 7 patients were lost, resulting in *n* = 27 patients with follow-up data. A participant flowchart is depicted in **Figure 1**. Recruitment was initially scheduled until November 2015. To achieve a higher sample size, it was extended until January 2016.

At baseline, the sample (*n* = 34) as displayed in **Table 2** was evenly distributed between groups (17 each) and consisted of 50% females. Mean age was 38.9 years (±11.3) at baseline. Three patients had a diagnosis of bipolar depression, 17 recurrent unipolar depression, and 14 first unipolar depression. The mean HDRS17 at baseline was 21.5 (±3.6), and the mean BDI was 26.6 (±8.1). BMI at baseline was 24.7 kg/m^2^, resting (systolic and diastolic) BP was 126.4/78.6 (±16.0/ ±9.4) mmHg, and resting HR was 73.1 (±13.0) bpm. Based on ANOVAs, independent-samples median tests, Mann–Whitney U tests, and χ^2^ tests, no significant group differences were found at baseline in any of the study variables.

**Table 2:** Description of sample at baseline.

	Intervention group		Control group		Total sample			
*n*	17		17		34			
	*n*	*%*	*n*	*%*	*n*	*%*		χ^2^ Test
Females (%)	8	47.1	9	52.9	17	50.0		*P* = 1.000
Smokers (%)	5	29.4	9	52.9	14	41.2		*P* = 0.296
Normally distributed variables*								
	*M*	*SD*	*M*	*SD*	*M*	*SD*		ANOVAs
Age at baseline	40.6	(8.8)	37.2	(13.5)	38.9	(11.3)		*P* = 0.397
Age at first episode	35.7	(11.3)	28.9	(14.7)	32.2	(13.4)		*P* = 0.148
HDRS17	22.4	(4.2)	20.7	(2.8)	21.5	(3.6)		*P* = 0.191
BDI	27.8	(8.8)	25.4	(7.3)	26.6	(8.1)		*P* = 0.391
PSQI	11.4	(3.9)	12.1	(4.1)	11.8	(4.0)		*P* = 0.659
MTQ18	47.1	(8.5)	44.1	(6.6)	45.5	(7.6)		*P* = 0.272
FEPS Rumination	3.9	(0.7)	3.9	(0.7)	3.9	(0.7)		*P* = 0.966
FEPS Focussing	2.9	(0.7)	2.6	(0.8)	2.7	(0.8)		*P* = 0.292
SCL-90: GS	147.3	(43.3)	145.1	(53.5)	146.2	(48.1)		*P* = 0.898
SCL-90: GSI	35.7	(11.3)	28.9	(14.7)	32.2	(13.4)		*P* = 0.148
BMI	25.7	(5.7)	23.7	(4.8)	24.7	(5.3)		*P* = 0.271
Systolic BP	127.4	(16.3)	125.5	(16.0)	126.4	(16.0)		*P* = 0.737
Diastolic BP	79.4	(7.0)	77.8	(11.4)	78.6	(9.4)		*P* = 0.629
sBDNF	20.7	(7.5)	21.3	(8.8)	21.0	(8.1)		*P* = 0.842
TNF-alpha	13.9	(7.9)	14.0	(8.1)	14.0	(7.8)		*P* = 0.967
Non-normally distributed variables*								
	Median	Min; Max	Median	Min; Max	Median	Min; Max	Independent-Samples Median Test	Independent-Samples Mann–Whitney U Test
Duration of episode (weeks)	10	3; 52	13	4; 104	11	3; 104	*P* = 0.225	*P* = 0.231
Prior depressive episodes (#)	1	0; 5	1	0; 30	1	0; 30	*P* = 0.818	*P* = 1.000
Prior maniac episodes (#)	0	0; 3	0	0; 0	0	0; 3	*P* = 1.000	*P* = 0.786
IPAQ-SF MPA	15	0; 180	30	0; 360	30	0; 360	*P* = 1.000	*P* = 0.518
IPAQ-SF VPA	15	0; 240	15	0; 180	15	0; 240	*P* = 0.839	*P* = 0.790
IPAQ-SF MVPA	45	0; 360	75	0; 540	60	0; 540	*P* = 0.616	*P* = 0.736
Resting HR	72	52; 98	71	52; 94	72	52; 98	*P* = 1.000	*P* = 0.193
CAR: AUC—total	331	145; 575	304	135; 806	318	135; 806	*P* = 1.000	*P* = 0.919
CAR: AUC—basal	271	107; 537	260	34; 497	265	34; 537	*P* = 1.000	*P* = 0.518
CAR: AUC—net	40	0; 246	95	0; 408	64	0; 408	*P* = 0.170	*P* = 0.120

*Normal distribution was tested with Kolmogorov–Smirnov and Shapiro–Wilk tests. HDRS17, Hamilton Depression Rating Scale 17. BDI, Beck Depression Inventory. PSQI, Pittsburgh Sleep Quality Index. MTQ18, Mental Toughness Questionnaire. FEPS, Fragebogen zur Erfassung allgemeiner und spezifischer Persönlichkeitsmerkmale Schlafgestörter; English: questionnaire for general and specific personality traits of insomniacs. IPAQ-SF, International Physical Activity Questionnaire (Short Form). MPA, moderate physical activity. VPA, vigorous physical activity. MVPA, moderate to vigorous physical activity. BMI, body mass index. BP, blood pressure. HR, heart rate. SCL-90, Symptom Checklist-90. GS, global severity. GSI, Global Severity Index. sBDNF, serum brain-derived neurotropic factor. TNF-alpha, tumor necrosis factor-alpha. CAR, cortisol awakening response. AUC, area under the curve.

### Dropout Analysis

We conducted an analysis of baseline characteristics between completers, dropouts, and participants who were lost to follow-up (**Table 3**). We found no significant differences in demographic variables, smoking status, cardiovascular measurements, symptom severity (including various other questionnaires such as PSQI, MTQ, FEPS, and SCL-90), and biomarkers (sBDNF and CAR). However, based on independent-samples Kruskal–Wallis tests, there was a significant difference in self-reported physical activity prior to hospital admission: higher scores for vigorous physical activity (VPA) were found in the group of patients who were lost to follow-up (median = 97.5 min, 0–240 min, *n* = 7) compared to dropouts (median = 0 min, 0–30 min, *n* = 9) and patients who participated in all data assessments (median = 0 min, 0–180 min, *n* = 27). The same pattern of results was found for moderate to vigorous physical activity (MVPA), with the highest scores found among patients who were lost to follow-up (median = 142.5 min, 15–360 min, *n* = 7), followed by patients who took part in all three data assessments (median = 45 min, 0–540 min, *n* = 27) and dropouts (median = 0 min, 0–60 min, *n* = 9).

**Table 3:** Dropout analyses.

	Completers			Dropouts			Lost to follow-up						
Normally distributed variables*													
	*M*	*SD*	*n*	*M*	*SD*	*n*	*M*	*SD*	*n*	*F*	*P*		*η^2^*
Age at baseline	39.0	11.1	29	40.2	11.9	13	36.0	12.3	7	0.30	0.742		0.013
Age at first episode	32.6	12.7	28	25.2	10.0	9	28.3	15.5	7	1.26	0.296		0.058
HDRS17	20.5	3.4	29	18.5	5.9	11	22.6	7.4	7	1.54	0.225		0.065
BDI	26.1	7.9	29	24.6	9.3	10	23.7	12.9	7	0.25	0.782		0.011
PSQI	11.4	4.1	28	8.6	3.4	9	13.5	2.6	6	2.99	0.062		0.133
MTQ18	45.8	8.0	28	46.5	12.3	10	47.9	7.9	7	0.14	0.866		0.007
FEPS Rumination	4.0	0.7	28	3.9	0.6	10	3.6	0.8	7	0.78	0.465		0.036
FEPS Focussing	2.7	0.8	28	2.9	1.0	10	2.5	0.8	7	0.47	0.632		0.022
SCL-90: GS	148.4	49.8	29	158.3	53.9	8	127.8	58.4	6	0.61	0.547		0.030
SCL-90: GSI	1.7	0.5	29	1.8	0.6	8	1.4	0.6	6	0.64	0.534		0.031
BMI	24.7	5.4	29	27.1	3.8	4	23.8	4.4	7	0.52	0.599		0.027
Systolic BP	125.7	16.9	29	123.3	11.8	4	128.6	16.3	7	0.15	0.866		0.008
Diastolic BP	78.5	11.0	29	82.3	13.7	4	74.4	4.7	7	0.76	0.473		0.040
sBDNF	21.9	8.5	29	25.6	9.6	12	22.6	12.1	7	0.65	0.529		0.028
TNF-alpha	13.5	7.7	29	7.9	7.3	13	13.6	9.1	7	2.49	0.094		0.098
Non-normally distributed variables*													
										Independent-Samples Median Test		Independent-Samples Kruskal–Wallis Test	
	*Median*	*Min; Max*	*n*	*Median*	*Min; Max*	*n*	*Median*	*Min; Max*	*n*	*P*		*P*	
Duration of episode [weeks]	12	3; 104	28	10	2; 100	9	9	4; 52	7	0.374		0.662	
Prior depressive episodes [#]	1	0; 30	28	3	0; 13	9	1	0; 3	7	0.264		0.409	
Prior maniac episodes [#]	0	0; 30	28	0	0; 3	9	0	0; 0	7	0.513		0.521	
IPAQ-SF MPA	15	0; 360	29	0	0; 60	9	30	0; 180	7	0.875		0.534	
IPAQ-SF VPA	0	0; 180	28	0	0; 30	9	98	0; 240	6	0.067		**0.034**	
IPAQ-SF MVPA	45	0; 540	28	0	0; 60	9	143	15; 360	6	0.218		**0.048**	
Resting HR	72	52; 98	29	67	61; 91	4	71	52; 94	7	0.810		0.911	
CAR: AUC-total	331	135; 806	29	319	86; 637	12	286	216; 366	7	0.437		0.750	
CAR: AUC-basal	271	87; 537	29	192	56; 713	12	174	34; 296	7	0.675		0.144	
CAR: AUC-net	57	0; 408	29	100	0; 210	12	93	0; 252	7	0.675		0.710	

*Normal distribution was tested with Kolmogorov–Smirnov and Shapiro–Wilk tests. All analyses (ANOVAs) are based on baseline scores. Completers, patients who have participated in all three data assessments (baseline, post, follow-up). Dropouts, patients who have only participated in the baseline data assessment. Lost to follow-up, patients who have participated in the baseline and postassessment but not in the follow-up assessment. BP, blood pressure. HR, heart rate. BMI, body mass index. HDRS17, Hamilton Depression Rating Scale 17. BDI, Beck Depression Inventory. PSQI, Pittsburgh Sleep Quality Index. MTQ18, Mental Toughness Questionnaire (18 items). FEPS, Fragebogen zur Erfassung allgemeiner und spezifischer Persönlichkeitsmerkmale Schlafgestörter; English: questionnaire for general and specific personality traits of insomniacs. IPAQ, International Physical Activity Questionnaire (Short Form). MPA, moderate physical activity. VPA, vigorous physical activity. MVPA, moderate to vigorous physical activity. SCL-90, Symptom Checklist-90. GS, global severity. GSI, Global Severity Index. sBDNF, serum brain-derived neurotropic factor. TNF-alpha, tumor necrosis factor-alpha. CAR, cortisol awakening response. AUC, area under the curve. Bold: p < .05.

### Adherence to Intervention Protocol

Of the 17 participants in the AE group, 11 (64.7%) completed all 18 training sessions. Three patients missed one session, two participants missed two sessions, and one participant only completed 12 sessions. As shown in **Table 4**, mean HR during AE was 128.8 bpm (71.8% HRmax, targeted: 60–75% HRmax). Mean expended energy amounted to 16.4 kcal/kg/week (range: 13.8–17.7), coming very close to the targeted dose of 17.5 kcal/kg/week.

**Table 4:** Adherence to intervention protocol of the aerobic exercise group.

	*M*	*SD*	Median	Range		Skew	Kurt
**Min**	**Max**
Mean HR (bpm)	128.8	(8.1)	127.3	115.7	150.7	1.14	2.44
HR% (%HRmax)	71.8	(2.9)	71.0	65.5	76.6	−0.11	−0.27
Weekly exercise duration (min)	146.4	(37.5)	111.1	91.6	248.7	1.35	2.53
Weekly kcal	1247.1	(369.4)	1,227.7	776.5	2107.5	0.89	0.43
kcal/kg/week	16.4	(1.3)	16.8	13.8	17.7	−0.93	−0.38
Total trainings (#)	17.2	(1.5)	17.0	12.0	18.0	−2.86	9.24
Score on Borg scale	13.1	(1.7)	12.9	10.1	16.6	0.34	−0.31

HR, heart rate. HRmax, maximal heart rate. kcal, kilocalories. kg, kilograms.

### Handling Missing Data due to Follow-Up

Given that a) nine participants dropped out from the baseline to the post-assessment and that b) seven participants were lost from the post to the follow-up assessment, we will carry out all analyses without and with intention to treat (45) when testing the effects of the intervention on specific outcomes. When analyzing the data without intention to treat, all participants with missing data will be excluded list-wise. Thus, only participants with valid data across all three measurement time points will be retained in the analyses. In addition to that, we will reanalyze the data with intention to treat, using a) multiple imputation and b) last observation carried forward (LOCF). While LOCF is considered the most conservative strategy to handle missing data, multiple imputation is a statistical technique for replacing missing data based on available information, hereby replacing each missing value with a series of plausible values.

## Discussion

The design of our study, incorporating an active placebo control (stretching) and applying a weekly exercise dose of 17.5 kcal/kg, is similar to that of existing studies with depressed inpatients (7, 10), allowing a certain comparability of our findings. However, the intervention period of 6 weeks is considerably longer than in most other inpatient studies (2–3 weeks), which may reflect the fact that inpatient treatment for severe depression lasts 6 weeks or more in German-speaking regions (46).

With a dropout rate of 20%, we lost more participants than previously found in a meta-analysis on dropout rates in exercise trials for depression conducted by Stubbs et al. (47). In their study, they found baseline depressive symptoms to predict greater dropout rates, which we could not replicate in our study. One possible explanation is that our study focused on a relatively homogeneous sample of inpatients with relatively severe depressive symptoms. In our study, patients with higher self-reported VPA levels prior to hospital admission were more likely to complete the study. This indicates that patients with low initial physical activity levels may need special support and motivational counseling to adhere to an AE program implemented as part of inpatient treatment.

Those participants who completed the AE intervention program generally showed adequate compliance, with 65% completing all 18 training sessions. Whereas the mean HR during training sessions was in the targeted range, we acknowledge that not all patients were able to reach the prescribed weekly exercise dose. Nevertheless, the mean expended energy (16.4 kcal/kg/week) was very close to the public health dose (17.5 kcal/kg/week) suggested by Dunn et al. (26). In summary, our manipulation check suggests that a reasonable degree of adherence to the planned intervention was reached in our sample.

Methodological strengths are an active control group, inclusion of two study centers, rigorous inclusion and exclusion criteria, clearly defined medical strategies, accurate monitoring of intervention implementation, and, as shown in the present paper, good adherence of patients to the study protocol. Despite these strengths, several limitations may influence the generalizability of the results. Possible limitations are the relatively short duration of the intervention, the small sample size, and the dropout of 20% of the participants. Given the substantial dropout rate from baseline to follow-up, examining intention-to-treat effects is needed in order to interpret the findings and to ensure that the analyses have the required power to find at least moderate effects of the intervention. Moreover, in the present study, the intervention period was determined by the typical length of patient hospitalization for severe depression in Switzerland. Although one might speculate that AE training as an add-on would produce stronger effects when the duration of hospitalization is longer, it is important to notice that in our investigation, AE was applied for a considerably longer period of time than in most previous inpatient studies (7, 9–11). Nevertheless, given that the duration of hospitalization varies across countries, we admit that this fact potentially limits the generalization of our findings. Furthermore, in light of the fact that participants with low initial physical activity levels were more likely to drop out during the intervention phase, we emphasize that this specific group of patients needs more support in future trials, for example, by establishing coaching mechanisms to address possible barriers of depressed patients toward exercise (48), by taking into consideration personal preferences with regard to the type and mode of physical activities (49), or by using graded exercise to slowly increase duration and intensity of the exercise regimen (50). Moreover, we acknowledge that we have included several secondary outcomes in our study. Given that these variables might be substantially correlated with each other, Bonferroni–Holm corrections are necessary to avoid the risk of alpha error inflation. For future studies, we also suggest to include a valid measure of VO_2_max and to rely on a multicenter design, resulting in a larger sample size. Finally, as shown by Hoffman et al. (51), it is possible that potential effects of an (short-term and structured) exercise intervention may dissipate after discharge from the clinics if participants are unable to maintain the exercise regimen. Therefore, studies examining the potential of intervention programs that contributed to sustainable improvements of physical activity behavior and physical active lifestyles (e.g., through individually tailored physical activity counseling) are highly needed. Another limitation may be the fact that patients with bipolar depression were included in the study (*n* = 3). Due to neurobiological differences, it could well be stated that exercise has different effects in unipolar compared to bipolar depression. However, a recent review has shown that patients suffering from bipolar depression profit from a wide range of beneficial effects of AE (52).

In conclusion, our study will add evidence on the effects of AE as an add-on to inpatient treatment of moderate to severe depression. Besides antidepressant effects, our study will shed light on potentially beneficial effects of AE on cognitive variables, sleep, HPA-axis activity, BDNF, TNF-alpha, and reactivity to psychosocial stress. Despite achieving fairly good adherence to the intervention protocol, the dropout rate suggests that depressed inpatients may need special support to adhere to a structured exercise intervention program.

## Ethics Statements

Ethical approval was obtained by the following ethics committees: i) Ethics Committee of Both Basel (EKBB) in Basel, Switzerland (reference no. 62/13, obtained on May 6, 2013), and ii) Ethics Committee Aargau/Solothurn in Aarau, Switzerland (reference no. 2013/029, obtained May 21, 2013). Today, both ethics committees are represented by the newly formed Ethics Committee of Northwest and Central Switzerland (EKNZ) in Basel, Switzerland. To assess an inflammatory parameter, we added measurement of TNF-alpha after completing the study; therefore, we obtained a protocol amendment by the EKNZ (reference no. PB_2016-02488, obtained November 14, 2016). The study was retrospectively registered on ClinicalTrials.gov in February 2016 (identifier: NCT02679053). All patients signed an informed consent to the study after receiving all relevant study information. Consent was obtained from the participants, collaborators, and co-authors.

## Author Contributions

CI, MG, JB, AE, UP, EH-T, and MH developed the study design. CI and JB coordinated the study at the two study sites. AE coordinated all laboratory analyses. CI and MG were responsible for data assessment and conducted the statistics. CI wrote the manuscript. All authors contributed to the data interpretation and the internal revision of the manuscript draft. All authors approved the final draft version.

## Funding

This study was financed by grants from the Gottfried and Julia Bangerter-Rhyner Foundation, the Helsana health insurance company, and the canton of Solothurn. The funding institutions took no part in the design of the study; in the collection, analysis, and interpretation of data; or in writing the manuscript.

## Conflict of Interest Statement

The authors declare that the research was conducted in the absence of any commercial or financial relationships that could be construed as a potential conflict of interest.

## Abbreviations

AE, aerobic exercise; ANOVAs, analyses of variance; BDI, Beck Depression Inventory; BDNF, brain-derived neurotropic factor; BMI, body mass index; BP, blood pressure; CAR, cortisol awakening response; FEPS II, Fragebogen zur Erfassung allgemeiner und spezifischer Persönlichkeitsmerkmale Schlafgestörter; English: questionnaire for general and specific personality traits of insomniacs; HDRS17, Hamilton Depression Rating Scale 17; HPA, hypothalamic–pituitary–adrenal; HR, heart rate; HRmax, maximal heart rate; ICD-10, International Classification of Diseases, Tenth Revision; IPAQ-SF, International Physical Activity Questionnaire; kcal, kilocalories; LOCF, last observation carried forward; MPA, moderate physical activity; MTQ18, Mental Toughness Questionnaire (18-item version); MVPA, moderate to vigorous physical activity; PSDQ, Physical Self-Description Questionnaire; PSG, polysomnography; PSQI, Pittsburgh Sleep Quality Index; sBDNF, serum brain-derived neurotropic factor; SCL-90R, Symptom Checklist-90; TAP, Testbatterie zur Aufmerksamkeitsprüfung; English: battery of tests for the assessment of attention; TAU, treatment as usual; TNF-alpha, tumor necrosis factor-alpha; TSST, Trier Social Stress Test; VPA, vigorous physical activity.

## Supplementary Material

Figure S1 G*Power Screenshot
