# Supplementary figures and images for: Effects of Aerobic Exercise as Add-On Treatment for Inpatients With Moderate to Severe Depression on Depression Severity, Sleep, Cognition, Psychological Well-Being, and Biomarkers: Study Protocol, Description of Study Population, and Manipulation Check
Source: Front Psychiatry. 2019 Apr 25;10:262. doi: 10.3389/fpsyt.2019.00262 (PMC6497035; doi:10.3389/fpsyt.2019.00262)

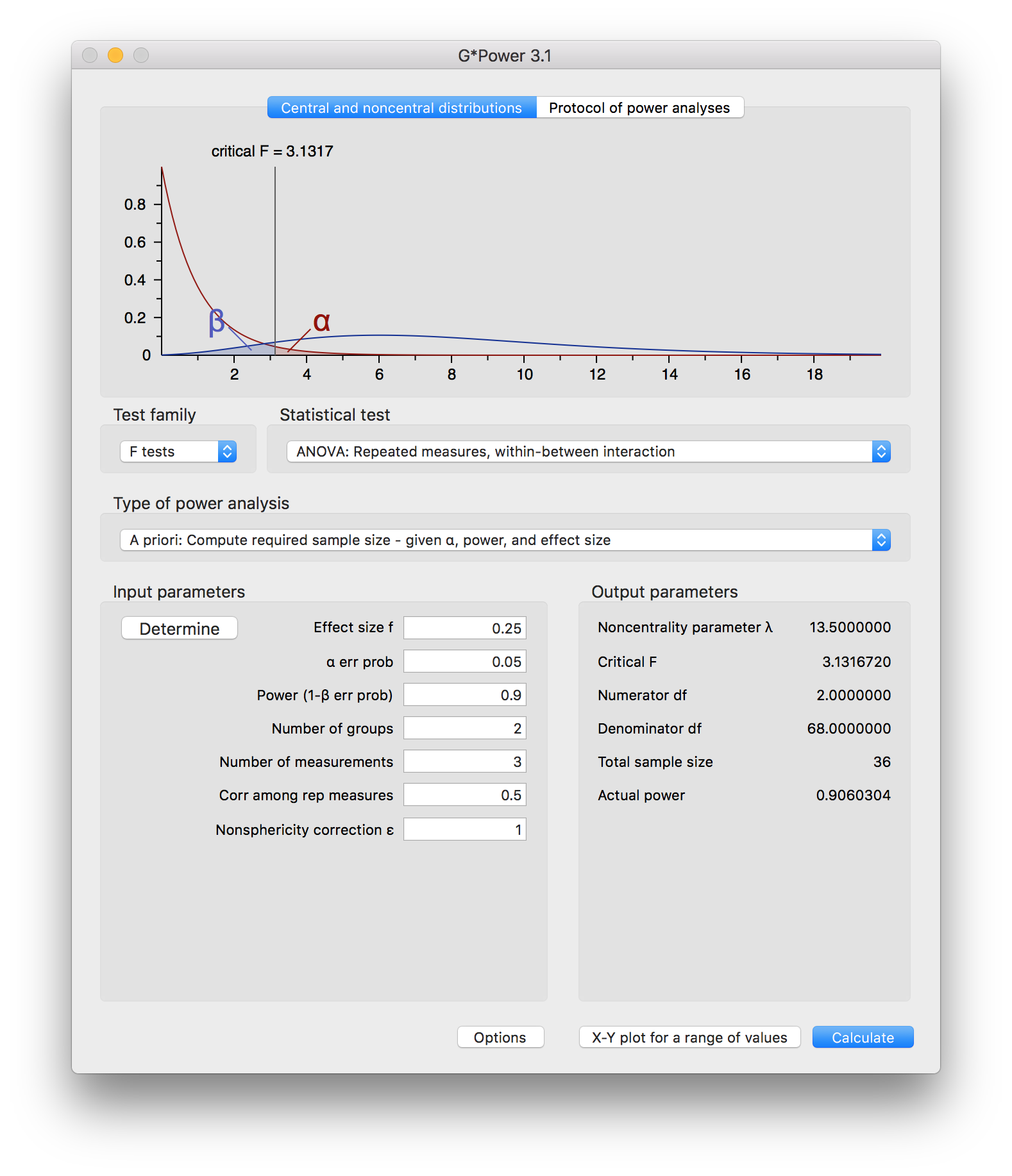

Supplement: Figure S1 — G*Power Screenshot [file Image_1.png]
